# Maternal physiological calming responses to infant suckling at the breast

**DOI:** 10.1186/s12576-023-00860-w

**Published:** 2023-03-14

**Authors:** Nami Ohmura, Lana Okuma, Anna Truzzi, Gianluca Esposito, Kumi O. Kuroda

**Affiliations:** 1 Laboratory for Affiliative Social Behavior, RIKEN Center for Brain Science, 351-0198 Saitama, Japan; 2 Laboratory for Human Cognition and Learning, RIKEN Center for Brain Science, Saitama, 351-0198 Japan; 3 Department of Psychology and Cognitive Science, University of Trento, Rovereto, TN Italy; 4 School of Psychology, Trinity College Institute of Neuroscience, Trinity College Dublin, Dublin, Ireland

**Keywords:** Lactation, Heart rate variability, Mother–infant interaction, Suckling, Milk ejection

## Abstract

The mother–infant relation is key to infant physical, cognitive and social development. Mutual regulation and cooperation are required to maintain the dyadic system, but the biological foundation of these responses remains to be clarified. In this study, we report the maternal calming responses to infant suckling during breastfeeding. Using behavioral measures and a Holter electrocardiogram as a readout of the maternal autonomic nervous system, the maternal activities during resting, sitting with her infant on her lap, and breastfeeding were assessed. We found that during breastfeeding, mothers talked less and maternal heart rate was lower than during sitting with the infant without breastfeeding. Congruently, maternal heart rate variability measurements indicated a higher parasympathetic activity during breastfeeding. Time-locked analyses suggested that this maternal calming response was initiated by the tactile stimulation at the breast by the infant face or mouth latch, which preceded the perceived milk ejection. These findings suggest that somatosensory stimuli of breastfeeding activate parasympathetic activity in mothers. Just as how the infant Transport Response facilitates the carrying of infants, the maternal calming responses during breastfeeding may promote efficient milk intake by inhibiting spontaneous maternal activities.

## Introduction

Mother–infant relations are critical for infant development in all mammals. Thus, both mothers and infants actively approach, act on and respond to each other. Breastfeeding is a prime example of such mother–infant interactions and it consists of a series of actions and reactions; typically, infants take the initiative in starting a breastfeeding bout, by emitting distress vocalizations to attract maternal attention. The mother approaches the infant in response, and the infant searches around and exhibits rooting to locate the nipple with the mouth. The mother takes a nursing posture, allowing the infant to latch on the nipple and suckle. The somatosensory stimuli by suckling are transmitted through the maternal spinal cord, the solitary tract nucleus, and then to the hypothalamus to induce pulsatile burst firing of oxytocin neurons [18, 40]. The released oxytocin in maternal peripheral circulation reaches the mammary gland, evoking milk ejection from her nipple. Then the infant starts vigorous and rhythmic suckling and swallows the milk. Gastric distention by milk ingestion has been shown to elevate plasma oxytocin in the offspring in animal models, which possibly induces satiety and positive psychological effects [23, 25, 31]. This chain of actions and reactions involves behavioral and physiological functions on both sides, but the detailed mechanisms of each component need to be clarified.

Lactation is known to influence general maternal states of affect, mood, and stress responses [14, 17]. Breastfeeding mothers exhibit relatively stronger parasympathetic dominance of the autonomic system, compared to non-breastfeeding mothers [22], and their cortisol level is decreased compared to bottle-feeding mothers, which indicates lower stress [12, 33]. However, previous investigations on the effects of each breastfeeding bout have mainly focused on the plasma hormone levels such as cortisol, oxytocin, and prolactin [2, 6, 37, 41], and leaving the acute physiological changes that happen during infant suckling understudied.

The current study stemmed from a case study assessing maternal stress factors during her daily activities, which inadvertently illustrated that the maternal heart rate decreased at each breastfeeding bout. Thus, we conducted a proof-of-concept experiment to examine the maternal behavioral and physiological changes while she interacted with her infant with and without breastfeeding.

## Methods

### Participants

All experiments were approved by the Ethical Committee of RIKEN (Japan) and was in accordance with the Declaration of Helsinki. Twenty-four healthy mothers with a healthy biological child who can breastfeed were recruited through advertisements and personal acquaintance. The ethnic groups of the participants were Japanese, French, and Finnish. Experiment sessions were conducted in either the participant’s home or at a laboratory setting, as they preferred. All mothers were fully informed about the contents of the study and gave written informed consent before the experiments. Details of participant information are summarized in Table 1.

**Table 1:** Participant information

Elapsed time since last breastfeeding for mothers			/26
	1–2 h	11.5%	3
	2–3 h	26.9%	7
	3–4 h	23.1%	6
	4–5 h	19.2%	5
	> 5 h	19.2%	5
Elapsed time since last suckling for infants (excluding solids)			/26
	1–2 h	11.5%	3
	2–3 h	30.8%	8
	3–4 h	26.9%	7
	4–5 h	15.4%	4
	> 5 h	15.4%	4
Infant condition			/26
	Teething	42.3%	11
	Started on solids	65.4%	17
Mother condition			/26
	Menstrual resumption	30.8%	8
Usual breastfeeding situation			/26
Breast milk or Formula	Only breast milk	53.8%	14
	Both	46.2%	12
Frequency of mother perceiving milk ejection			/26
	Most times	30.8%	8
	Sometimes	42.3%	11
	No	19.2%	5
	Don't know	7.7%	2
Presence of perceived milk ejection in experimental session			/26
	In session	76.9%	20
	In first-round *BF*^+^	76.9%	20
	In second-round *BF*^+^	23.1%	6
	Both two round	23.1%	6
	No	23.1%	6
Presence of perceived milk ejection in analyzed 3-min period			/23
	In analyzed period	17.4%	4
	In the same side except for the analyzed period	47.8%	11
	Only in the opposite side	8.7%	2
	No	26.1%	6

Data were obtained from a pre-experimental questionnaire and from the actual carryout of the experiment

### ECG recording and extraction of IBI

Electrocardiogram (ECG) signal was acquired using a portable Holter (at 1024 Hz, CardioMem® CM 4000, GETEMED) with 2 channels throughout the experimental session. A total of five ECG electrodes (Ambu® BlueSensor N, Ambu) were placed on the back of the mother, so that they do not impede with the recording while nursing or while the mothers interacted with her infant at her front. ECG data from the Holter were preprocessed using the CardioDay® software (GETEMED) and a python program, which identified the QRS complexes and extracted a list of inter-beat-intervals (IBIs), which is the time elapsing between two consecutive R waves. IBI extraction errors were fixed by visual inspection and calibration. Manual correction of IBIs happened in 267 Rs (1.65%) out of the 16150 IBIs of all the relative data.

The ECG data and recorded video were synchronized by pressing the event button on the Holter to mark the time during ECG recording. Synchronization and behavioral coding were done at each IBI level, using the software Premiere Pro CS5 (Adobe) or VideoPad (NCH Software).

### A case study of day-long maternal ECG

One female participant (age = 34) was recorded of her ECG during her natural daytime behavior at home over seven hours. Behaviors were recorded with a video camera from one corner of the ceiling. The original purpose of this observation was to assess maternal psychological stress during interactions with her family members. As such, she followed her daily routine including housework (cooking, washing dishes, folding the laundry), eating lunch with her family, deskwork in a sitting position (household accounting, filling in documents), caring for her children including breastfeeding her son (5 month-old), and talking with her husband and daughter (age = 3). At the beginning and end of the recording, she was instructed to perform an additional 10 min of resting and 10 min of calculation while seated alone as a control condition to assess the effects of psychological stress on the IBI in the sitting condition. Long IBI plots over 7 h were plotted by removing presumed erroneous IBIs that deviated by more than ± 150 ms from each mean of the nearby 40 beats.

### Participants in the breastfeeding experiment

Data were obtained from 26 mother–infant dyad. This included three mothers who participated twice with her subsequent child. The mean maternal age was 34.83 years (SD = 4.53, SEM = 0.92, Min = 28, Max = 42). The mean infant age was 6.98 months (SD = 3.95, SEM = 0.77, Min = 2.03, Max = 22.13, all but one were under 1 year old), with 16 boys and 10 girls. Because the IBI property of the mother whose infant age was over 1 year did not differ from the others, her data were also included in the analysis. Experiment location was at the participant’s home for 9/26 (34.62%), and at a laboratory setting for 17/26 participants (65.38%). Prior to their participation, all the mothers completed a questionnaire about the infant’s birthday, sex, birth order, teething, solid food, and about the mother’s age, her usual breastfeeding style, and how often she felt milk ejection. None of the mothers reported to be pregnant at the time of experiment, and no one had experienced any heart disease nor abnormalities. Before starting the experiment, mothers answered the state of health for the dyad for that day, as well as reported how many hours it had been since she last breastfed her infant. Participants except for laboratory staff (84.62%, 22/26) were informed of the experimental purpose and task details, and not of the experimental hypothesis (i.e., IBI changes during breastfeeding) prior to the experiment. Six of the mothers were subject to a simultaneous electroencephalogram (EEG) recording in addition to the ECG, of which results are not addressed in this article.

### Maternal experimental tasks

Mothers performed the following tasks while seated on a chair without leaning against the backrest throughout the experimental session. For the first five minutes, the mother was instructed to sit alone with her eyes open, to take a few deep breaths, and to refrain from talking as much as possible (*resting*). During this condition, the infant was separated from the mother and was kept in the same room by an experimenter, who showed toys and soothed the crying infant if necessary. Secondly, the infant was returned to the mother’s lap and the dyad interacted for another five minutes (*with infant BF*^−^), while the mother was allowed to talk. Sometimes, the infant was allowed to hold a toy. Any gestures from the infant to be breastfed was gently declined by the mother. Then, upon the experimenter’s cue, the mother prepared and started to breastfeed the infant (*with infant BF*^+^). The infant could suckle on one side of the breast for as long as and as many times as it wanted. In order to record the moments of attachment and detachment of the infant’s mouth to the mother’s nipple, the mother reported these either orally or by raising her hand. Participants were also asked to report the moments when she perceived milk ejection, as they were informed in advance that examples of such a perception may be a tingling sensation at breast, feeling a rapid increase of milk flow, or milk dripping from the other breast. If the infant seemed to have fallen asleep while breastfeeding, or if the suckling duration was beyond five minutes, the mother could take her nipple out of the infant mouth. The *BF*^−^ and *BF*^+^ conditions were repeated for another round with her other breast.

All sessions were videotaped either by a handy camera or from a fixed position in the front. The video always contained at least the mother’s upper half of body, and the infant’s whole body when it was being held on the mother’s lap.

### Data selection for comparative analysis between three conditions

The videos were coded for the experimenter’s cues at the start and stop of each task, and when other events occurred, such as when the mother reported the latch-on and detachment of the infant’s mouth to the nipple, all at a resolution of each maternal IBI bin. Each condition was defined to be the time between the experimenter’s cues in the conditions *resting* and *with infant BF*^−^, and also to be between the latch-on and detachment of the infant’ mouth to the nipple in *with infant BF*^+^. Three-minute periods of data from the middle of each condition were used for the frequency-domain analyses to avoid initial body movement effects and other likely transition effects due to the switching of conditions. In the *BF*^+^ condition, infants occasionally let go of the nipple before 3 min had passed, therefore, the 3-min data were extracted from the first segment in which the continuous attachment to the nipple exceeded 3 min.

The *BF*^−^ and *BF*^+^ data from each participant were always a successive pair, drawn from either the first round or the second round of the *with infant* conditions. If 3 min of data were not available for all of the three conditions, the participant was excluded from the condition comparison analysis, resulting in 23 participants eligible for this analysis.

### Maternal and infant vocalization analyses

Maternal vocalization and infant negative vocalization were coded at each IBI bin for their presence or absence, and then the proportion of time spent vocalizing was calculated for the 3-min periods described above. In the analysis of maternal vocalization, vocalization property was also classified by whether it was directed towards the infant or towards the experimenter, and if so, whether her vocalization was spontaneous or induced such as a reply, a reaction, or an obligatory report. Maternal spontaneous vocalization was defined as all the vocalizations except for the induced ones towards the experimenter. The mothers were instructed not to talk during the *resting* condition, although some mothers still vocalized during this period.

### Maternal sleepiness score

Scores of self-reported maternal sleepiness were drawn from a post-experiment questionnaire. Mothers were asked to recall her level of sleepiness with the 4-scale score in each condition, as well as whether there was anything disturbing in the environment with yes/no answer. The options for the level of subjective sleepiness were: 0, did not feel sleepy; 1, spaced out a little; 2, fell into a doze; and 3, almost fully asleep.

### Analysis of heart rate variability

The heart rate variability (HRV) of the 3-min IBI data were analyzed using a Python program. As time-domain indices, the mean IBI, RMSSD (root mean square of successive R–R interval differences, reflecting beat-to-beat variance in IBI), and pNN50 (percentage of successive R–R intervals that differ by more than 50 ms, correlated with parasympathetic nervous system activity) were calculated. In the frequency-domain indices, fast-Fourier transform was applied after interpolation, normalization, and windowing with a Hamming-window to the IBI data, providing power distribution values at very low-frequency (VLF, 0.00001 – 0.04 Hz), low-frequency (LF, 0.04 – 0.15 Hz), and at high-frequency (HF, 0.15 – 0.4 Hz). These were then normalized by dividing each with the sum of VLF + LF + HF, to calculate LFnu (relative power of the low-frequency band in normalized unit, reflecting baroreceptor activity during resting conditions), HFnu (relative power of the high-frequency band in normalized unit, an indicator for parasympathetic activity and corresponds to the IBI variations related to the respiratory cycle), and LF/HF ratio (the ratio of LF to HF power, which estimates the balance between sympathetic and parasympathetic nervous systems).

### IBI transition by microevent

The influence of behavioral microevents around breastfeeding initiation on maternal IBIs was analyzed as follows. Videos were coded with an IBI level precision for the moment when the infant’s face touched the maternal breast directly, when the mother reported the first latch-on, and when the mother reported perceiving milk ejection. Peri-event IBIs (± 20 heartbeats from the microevent) were aligned to the moment of event initiation as heartbeat count = 0, and were normalized by subtraction to the mean IBI at count 0. Normalized IBIs (nIBIs) at each heartbeat before and after the microevent were compared to the baseline nIBI at count 0.

### Statistical analyses

All statistical analyses were conducted using SPSS Statistics 23 and R version 4.1.0.

Shapiro–Wilk test for normality, Levene’s test for variance, Friedman test, paired t-test, Wilcoxon signed-rank test, Student’s t-test, Welch’s t-test, Mann–Whitney U test, Steel’s test, Spearman’s correlation coefficients and RM ANOVA were used. Multiple comparisons were conducted with *p*-values adjusted by Benjamini–Hochberg’s method.

An analysis of the effects of between-subject variables, including maternal and infant age, infant sex and birth order, experimental location (either at home or in the lab), and whether the data pair was drawn from the first or the second round of *BF*^−^/*BF*^+^, did not reveal any main effects on maternal IBI or HRV.

## Results

### Maternal stress, breastfeeding and heart rate measurements

**Fig. 1:**
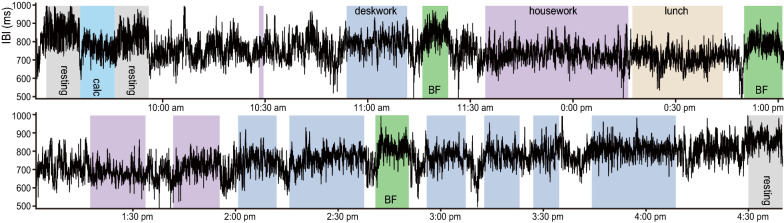
Maternal IBI transition during her daily home activities. One mother’s IBI plot during her natural daily activities at home for about 7 h. At the beginning and end of the experiment, the mother performed resting, math calculation and resting for 10 min each while seated. Shaded areas indicate her behavioral tasks of resting (grey), calculation (light blue), deskwork (dark blue), breastfeeding (*BF*, green), housework (purple), and lunch (brown)

The present study was prompted by an inadvertent finding of the calming effect of breastfeeding, during a trial experiment to evaluate the psychological stress of a mother. Maternal behavior was monitored by video recording, and maternal cardiac physiology using a Holter electrocardiogram (ECG) (Fig. 1). We aimed to infer the maternal stress by inter-beat interval (IBI), which is the time interval between consecutive heartbeats obtained from the ECG. Then her behaviors and IBI were recorded during her daily home activities, including cooking, eating lunch with her family, talking with her husband, and interacting with her 3-year-old child and her 5-month-old infant, caring for the children, and breastfeeding the infant. In order to control the effects of physical and psychological activities on the IBI, the mother was asked to have extra periods of sitting still while quietly resting or desk work for household accounting. The results showed that, over the 7-h recording, maternal IBI during each period of breastfeeding appeared to be higher than the IBI during desk work, and it was as high as the IBIs in the resting condition (Fig. 1). This observation led us to hypothesize that mothers are in a relatively relaxed state during breastfeeding, not only because it involved sitting still.

### Outline of the experimental design

**Fig. 2:**
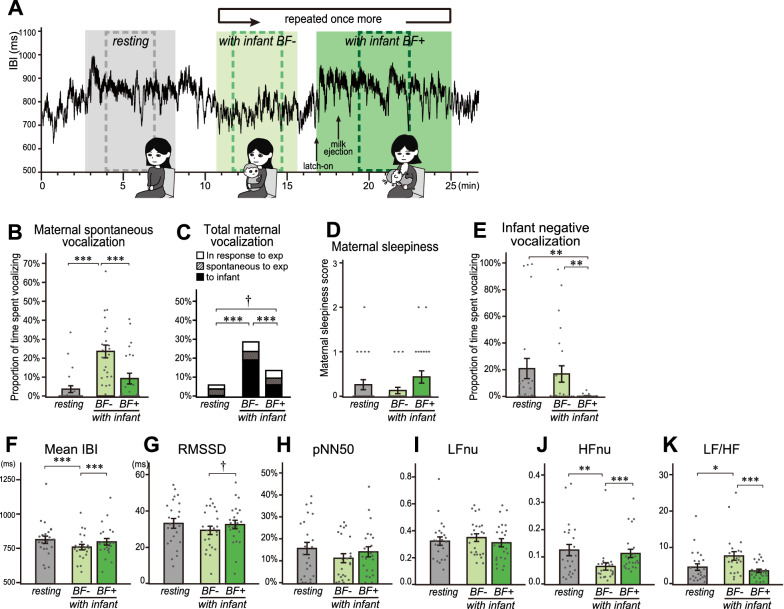
Breastfeeding-induced calming responses in mothers. A 3-min data from the middle of each condition for 23 of 26 participants were used for this analysis. **A** The schema for the breastfeeding experiment and an example IBI plot. Mothers sat alone for 5 min (*resting*, grey), sat with her infant on lap for 5 min (*BF*^−^, light green), breastfed from one side for some time (*BF*^+^, dark green), and repeated the *with infant* conditions once more. Vertical arrows indicate the timings when the mother reported the infant’s latch-on and when reported perceiving the milk ejection. IBI and vocalization data in the middle 3 min of these conditions (dashed boxes) were analyzed. **B** The proportion of the amount of maternal spontaneous vocalization by condition, excluding her replies and reports towards the experimenter. **C** Proportion of maternal vocalization and its subdivision to the duration of each condition. Each vocalization was categorized by whether it was directed towards the infant or the experimenters, and if the latter, whether it was spontaneous or induced. In **B**, the sum of “spontaneous to exp” and “to infant” is shown. **D** Maternal sleepiness scores for each condition, from a post-experiment questionnaire. At the end of the experiment, mothers recalled and chose her level of sleepiness from a range between not sleepy (score: 0) and difficult to stay awake (score: 3) for each condition. **E** Proportion of infant negative vocalization to the duration of each condition. **F–K** Heart rate variability measures in the time-domain (**F**–**H**) and the frequency-domain (**I**–**K**). Bars represent mean ± SEM and dots represent individual data points. Multiple comparisons were done by Friedman’s test, and a post hoc Wilcoxon signed-rank test for all pairs, with *p*-values adjusted by Benjamini–Hochberg’s method, †*p* < 0.1, *p < 0.05, **p < 0.01, ***p < 0.001

To directly test this hypothesis, 26 breastfeeding mothers who had infants were recruited, and data from 23 dyads with sufficient recording quality were used (Table 1). The behavioral experiment consisted of three conditions that were all performed while seated on a chair (Fig. 2A): (1) the mother rested alone with eyes open and was instructed to refrain from talking for five minutes (*resting*), (2) the mother held her infant on lap for five minutes (*with infant BF*^*−*^), and (3) the mother held her infant and breastfed from one side of the breast as long as the infant wanted (*with infant BF*^+^). The mothers were asked to report when the infant mouth latched on to the maternal nipple, and whenever a milk ejection reflex at the breast was perceived. Participants were informed in advance about examples of the perception of milk ejection. Subsequently, the conditions (2) and (3) were repeated for the other side of the breast. These second-round data were used when the breastfeeding was shorter than 3 min in the first-round. At the end of the experiment, mothers were asked to recall their level of sleepiness in each condition.

Video and ECG were recorded throughout the tasks. Then 3-min data were cut out from the middle of each condition and were analyzed for maternal and infant vocalizations, maternal sleepiness, IBI and heart rate variability (HRV) indices (Fig. 2A–K). HRV, which is the fluctuation in the time intervals between adjacent heartbeats [29], was evaluated as a readout of maternal autonomic function, utilizing the time-domain indices rMSSD (root mean square of successive R–R interval differences) and pNN50 (percentage of successive R–R intervals that differ by more than 50 ms), and frequency-domain indices LFnu (relative power of the low-frequency band 0.04 – 0.15 Hz in normalized unit), HFnu (relative power of the high-frequency band 0.15 – 0.4 Hz in normalized unit), and LF/HF ratio. The HFnu reflects the regulatory mechanism that control heart rate via respiration-driven acceleration and deceleration of the heart via the vagus nerve, termed the *respiratory sinus arrhythmia* and is regarded as an index of parasympathetic activity. The LF/HF ratio is an estimate for the balance between sympathetic and parasympathetic nervous system activities.

### Maternal calming responses during breastfeeding

The amount of spontaneous maternal vocalization was higher during *BF*^*−*^ than that during *BF*^+^, when they were not restricted to talk in these conditions (Fig. 2B, 2C). 46.2% (12/26) of the mothers reported to have felt sleepiness in one or both rounds of *BF*^*+*^ condition in a post-experiment questionnaire. The mean maternal subjective sleepiness appeared to be higher in *BF*^+^ than that in *BF*^*−*^, but it did not reach to the statistical significance (Fig. 2D). Interestingly, maternal sleepiness correlated positively with HFnu and negatively with LF/HF only during *BF*^*−*^ and not during other conditions (Table 2). These data suggested that breastfeeding calmed the maternal spontaneous vocalizations.

**Table 2:** Vocalization and sleepiness in breastfeeding mother and their relation to HRV indices

Correlation (*N* = 23)			*Resting*				*BF*^*−*^				*BF*^*+*^				*With infant* (*BF*^*+/ｰ*^)			
			M_sleepiness	M_totalVocal	M_spVocal	I_NegVocal	M_sleepiness	M_totalVocal	M_spVocal	I_NegVocal	M_sleepiness	M_totalVocal	M_spVocal	I_NegVocal	M_sleepiness	M_totalVocal	M_spVocal	I_NegVocal
Spearman's ρ	*IBI*	Correlation coefficient	0.191	0.226	0.292	0.002	− 0.019	0.000	− 0.034	− 0.045	− 0.212	− 0.036	− 0.052	0.233	− 0.224	− 0.112	− 0.068	0.165
		Sig.(2-tailed)	0.382	0.299	0.177	0.993	0.930	1.000	0.879	0.837	0.332	0.870	0.813	0.284	0.303	0.612	0.757	0.451
	*RMSSD*	Correlation coefficient	0.107	0.269	0.267	0.100	0.019	0.161	0.130	− 0.228	− 0.311	0.080	0.052	0.040	− 0.308	0.150	0.118	-0.102
		Sig.(2-tailed)	0.626	0.214	0.218	0.650	0.930	0.463	0.553	0.295	0.148	0.716	0.813	0.855	0.153	0.494	0.593	0.645
	*pNN50*	Correlation coefficient	0.138	0.192	0.178	0.038	− 0.010	0.172	0.165	− 0.204	− 0.348	0.109	0.065	0.075	− 0.313	0.142	0.121	-0.109
		Sig.(2-tailed)	0.531	0.381	0.417	0.864	0.965	0.433	0.453	0.351	0.103	0.621	0.769	0.735	0.146	0.517	0.584	0.621
	*LFnu*	Correlation coefficient	0.252	− 0.029	− 0.027	− 0.109	− 0.253	0.499^*^	0.399^†^	− 0.192	− 0.041	0.405^†^	0.501^*^	0.061	− 0.192	0.513^*^	0.430^*^	− 0.064
		Sig.(2-tailed)	0.247	0.895	0.903	0.621	0.244	0.015	0.059	0.380	0.852	0.055	0.015	0.781	0.381	0.012	0.041	0.772
	*HFnu*	Correlation coefficient	0.100	− 0.364^†^	− 0.428^*^	− 0.157	0.448^*^	− 0.145	− 0.264	− 0.068	− 0.129	− 0.222	− 0.093	− 0.232	0.052	− 0.206	− 0.291	− 0.197
		Sig.(2-tailed)	0.651	0.088	0.041	0.475	0.032	0.508	0.224	0.759	0.558	0.308	0.672	0.287	0.813	0.347	0.179	0.368
	*LF/HF*	Correlation coefficient	0.060	0.337	0.411^†^	0.079	− 0.545^*^	0.449^*^	0.513^*^	− 0.061	− 0.010	0.449^*^	0.463^*^	0.305	− 0.167	0.508^*^	0.507^*^	0.108
		Sig.(2-tailed)	0.786	0.116	0.052	0.719	0.007	0.032	0.012	0.783	0.964	0.032	0.026	0.157	0.447	0.013	0.014	0.624

Spearman’s correlation coefficients (*ρ*) and their *p*-values, calculated for the relationship between each of the four behavioral variables (M_sleepiness, maternal sleepiness; M_totalVocal, maternal total vocalization; M_spVocal, maternal spontaneous vocalization; I_NegVocal, infant negative vocalization) and each of the six dependent variables of HRV (mean IBI, RMSSD, pNN50, HFnu, LFnu, and LF/HF). The coefficients were calculated for each of three conditions, as well as for the combined *with infant* condition (*BF*^+^ and *BF*^−^) (rightmost column)

^†^*p* < 0.1, *p < 0.05

As for the HRV (Fig. 2F–K), IBI and HFnu were significantly higher in *BF*^+^ than those in *BF*^*−*^, and LF/HF was significantly lower in *BF*^+^ than that in *BF*^*−*^. In addition, there was no significant difference in all the HRV between *BF*^+^ and the *resting*. These data indicate that the maternal parasympathetic activity in *BF*^+^ was increased by breastfeeding to a similar level with that during *resting*.

### Evaluation of the effects of infant and maternal vocalizations as possible confounding factors

Infant negative vocalizations, which were significantly greater during *BF*^*−*^ than during *BF*^+^, may have been stressful for the mother (Fig. 2E). However, the infant negative vocalizations were even higher during the *resting* condition, nor did they ever correlate with IBI or any HRV indices (Fig. 2E; Table 2). Therefore, the effects of *BF*^+^ on HRV are unlikely to be caused by the decreased maternal stress inflicted by infant vocalizations.

Maternal vocalizations were also more frequent during *BF*^*−*^ than during *BF*^+^ and may have affected the IBI and HRV differences between these conditions. Indeed, maternal vocalizations correlated positively and consistently with LFnu and LF/HF during both of the *with infant* conditions (Table 2). However, it has been shown that speech causes longer respiratory cycle that affects LF range of HRV [3]. Considering that maternal vocalization did not correlate with IBI or the time-domain HRV indices in any conditions, it was inferred that the correlations between maternal vocalization and the LFnu and LF/HF were caused by the respiratory control during speech, rather than by the maternal autonomic activities.

Overall, these confounding factors did not appear to significantly disturb the above interpretation that breastfeeding had a calming effect similar to resting.

### Maternal calming precedes actual milk ejection

**Fig. 3:**
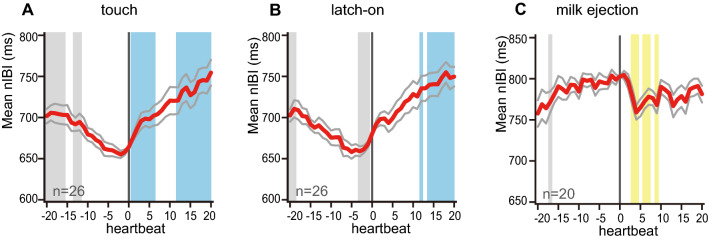
Maternal IBI transition at the initiation of breastfeeding and at the perceiving of milk ejection. Data from which each event timing could be extracted among the 26 participants were analyzed, when the infant’s face touched the mother’s breast (**A**), when the infant latched on to the nipple (**B**), and when the mother reported perceiving a milk ejection (**C**). Peri-event nIBIs are shown in mean (red lines) ± SEM (gray). Vertically shaded colors indicate significant differences (*p* < 0.05) compared to the nIBI at heartbeat = 0 (yellow, decreased after the microevent; blue, increased after the microevent; grey, higher or lower values before the microevent) by Steel’s test

We initially hypothesized that this maternal calming effect elicited by breastfeeding may be due to oxytocin, which is released from the neurohypophysis to peripheral blood circulation in response to mechanical suckling, and induces milk ejection by contracting the myoepithelium cells in the mammary gland. To test this hypothesis, we examined the IBI transitions aligned at three major events: when the infant face touched to the maternal breast, when the infant mouth properly held the maternal nipple (latch-on), and when the milk ejection was perceived by the mother (Fig. 3). Maternal IBIs were aligned at the initiation of each event as heartbeat count 0. Then the peri-event IBIs were normalized (nIBI) by the mean IBI at the count 0, and were averaged across the mothers at each count, for 20 heartbeats before and after the event. Surprisingly, the nIBI increase started when the infant's face first touched the maternal breast or the latch-on (Fig. 3A, B). This increase typically continued until the timing of the milk ejection, which occurred about 49.15 ± 6.69 s after the latch-on, by which the maternal mean nIBI reached a plateau of about 800 ms. In the 20/26 mothers who reported that they perceived a milk ejection, the milk ejection decreased her nIBI slightly but significantly during the subsequent 10 beats (7.79 s) (Fig. 3C). Maternal verbal reporting of the timing of milk ejection was unlikely to cause this tentative decrease in nIBI, because the mothers also reported the infant latch-on verbally and did not show such a decrease. These data suggested that the tactile stimulation to the maternal breast by the infant, rather than the increase of plasma oxytocin, could mediate the calming effect of the breastfeeding.

## Discussion

### Physiological calming in nursing mothers

This study showed that mothers during breastfeeding spontaneously reduce the amount of conversation and enter a relaxed state of parasympathetic predominance within a minute. Previous studies that examined the effects of breastfeeding on mothers have often focused on the general maternal state, comparing breastfeeding mothers to non-breastfeeding mothers in the broad time span of the lactation period, or focused on the hormonal change in maternal blood during breastfeeding [2, 22]. Our results provide sub-second physiological changes derived from maternal ECG, synchronized with observations of maternal behavior as she breastfed her infant. This result is similar to another previous case report with EEG, where they reported that breastfeeding increases the 6–10 Hz waves in the central–parietal and parietal–temporal derivations of the 10–20 international system, which they referred to as EEG signs of “relaxation behavior” [5]. Thus, the mothers are in a relaxed state not only during the broad lactation period, but also in the moments of each breastfeeding.

### Maternal sleepiness during breastfeeding

In many mammals, lactating mothers often assume a light sleep state during nursing and milk ejection. In rat studies, milk ejection is always preceded with an occurrence of slow-wave sleep in their EEG, suggesting that sleepiness may be a prerequisite for milk ejection [20, 39]. This is reasonable because most frequent breastfeeding occurs during the inactive nesting period for the mother; during light-phase (daytime) in nocturnal mice and rats, and during night in diurnal primates, the mothers are essentially drowsy/asleep and the infants suckle periodically throughout the nesting time [16, 19]. However, especially for large animals including humans, deep sleep during nursing is undesirable as the mothers may toss and turn over the infants. Maternal postural regulation and alertness to infant signals should be maintained at least during nursing, so that infants are not accidentally suffocated.

Human mothers may similarly feel drowsy while breastfeeding. Many human mothers are reported to be less-aroused, and in a positive mood with reduced stress and anxiety in their lactation period [12–14, 17]. In this study, however, we could not detect a statistically significant difference in subjective sleepiness between *BF*^*−*^ and *BF*^+^ (Fig. 2D). Nine out of 12 participants who reported sleepiness in *BF*^+^ were in a lab environment, suggesting that the experimental environment did not grossly affect the occurrence of sleepiness. However, several limitations of the present study, such as the fixed task order, the presence of experimenters, and the equipment of recording devices may have distracted the naturalistic responses in the breastfeeding mothers, and should be improved in future studies. Additional measurements such as EEG analysis, may also provide a better evaluation of drowsiness.

### Elevation of IBI with breastfeeding is caused by tactile effects, not by oxytocin effects

Oxytocin plays a major role in milk ejection during breastfeeding, and is also well known for its anxiolytic effects partly via the parasympathetic nervous system [37], although the presence of oxytocin is not essential for the nursing behavior per se [24, 32]. The present result indicates that the IBI elevation coincided with the initiation of somatosensory stimulus by touch or latch-on to the breast by the infant mouth, and that IBI rather decreased transiently at the timing of perceived milk ejection (Fig. 3), consistent with the previous literature reporting the increase of heart rate by oxytocin [27, 28]. Consistently, several reports suggest that infant's suckling stimulation to the mother's nipple excites the maternal vagus nerve via the somatosensory nerves innervating the nipple, reflexively stimulating the secretion of gastrointestinal hormones and inducing drowsiness in mammals including humans [35, 36]. Thus, the parasympathetic activation in breastfeeding seems not to be due to oxytocin release inducing milk ejection. As a caveat, our analysis did not formally exclude the possibility that oxytocin secretion begins prior to the onset of lactation by classical conditioning called "Pavlovian milk conditioning"; a previous report on blood oxytocin levels associated with breastfeeding have demonstrated that maternal oxytocin levels increased upon hearing the infant cry, which was far earlier than the onset of breastfeeding [8, 21]. However, in our study, 26.1% (6/23) of the infants were fussy or crying during *resting* or *BF*^−^, which always preceded *BF*^+^, but the mean nIBI only increased at the onset of *BF*^+^ (Fig. 3A, B). The mother–infant dyad shown in Fig. 2A also demonstrated that the maternal IBI increased at the onset of *BF*^+^, even though the infant was crying during *resting* and *BF*^−^. These observations collectively support the notion that the elevated IBI with breastfeeding is initiated by the tactile stimulation to the maternal breast. The limitation of this study was that we could not clearly differentiate which exact type of tactile stimulus caused the IBI elevation: whether it could be any infant contact to the breast, or it had to be the mouth firmly latching on to the nipple. Future studies may also include the group of bottle-feeding mothers to address the precise mechanism of maternal IBI changes by breastfeeding.

### Dysphoric Milk Ejection Reflex

Dysphoric Milk Ejection Reflex (D-MER), characterized by dysphoria starting shortly before milk ejection and progressing for several minutes, has been reported to have a prevalence of 9.1% [7, 34] in the population. Two mothers in our experiment reported such an unpleasant feeling in their usual breastfeeding, but their IBI changes were not particularly different from those of other mothers. D-MER is thought to be a psychological-based mechanism mediated by dopamine, and the mechanism may be different from the physiological IBI and HRV changes observed in breastfeeding.

### Turn-taking calming responses as an anti-phase synchrony between the mother–infant dyad

We have previously demonstrated that human infants and mouse pups can be calmed immediately by parental carrying and that it reduces their cry, spontaneous movements, and their heart rate, partly mediated by the parasympathetic activation [10, 26, 42, 43]. This evolutionarily conserved set of physiological and behavioral responses to carrying is termed the "Transport Response" and is widely observed across mammalian species, regarded as infant cooperative responses to parental carrying [4, 9]. This study describes a counterpart phenomenon for the lactating mothers being calmed during infant suckling activities. The calming response of breastfeeding mothers may well meet the goal of effectively feeding the infant.

These two lines of research propose a novel anti-phase synchrony of the mother–infant dyads. While many reports have identified several instances of in-phase synchrony in the mother–infants in emotional interactions like eye gaze, emotional regulations and verbal and non-verbal communications and social play [11, 30, 38] these passive "inactions" are interesting because it enables physiological and behavioral turn-taking: while the major actor of the dyad is engaging in a necessary duty for survival (such as transport or suckling), the counterpart should restrict their own activity and stay calm for a while, not to disturb the main actor. This type of inhibitory cooperation may not be as conspicuous as active role-taking and thus may have been understudied. Such a rapid physiological calming, induced by the partner’s activities, may play a crucial role as a “hidden regulator” of the mother–infant symbiosis [1, 15].

## Conclusions

The present study demonstrates the physiological calming effects of breastfeeding on lactating mothers. The state of parasympathetic dominance during breastfeeding appears to be initiated by the onset of breastfeeding via tactile stimulation, and it happens earlier than perceived milk ejection.

## Data Availability

The data generated in this study are available from the corresponding author upon reasonable request with a completed Materials Transfer Agreement, excluding the materials including personally identifiable information.
